# The Molecular Mechanisms and Therapeutic Targets of Atherosclerosis: From Basic Research to Interventional Cardiology

**DOI:** 10.3390/ijms25094936

**Published:** 2024-04-30

**Authors:** Josip Andelo Borovac

**Affiliations:** 1 Division of Interventional Cardiology, Cardiovascular Diseases Department, University Hospital of Split (KBC Split), 21000 Split, Croatia; jborovac@mefst.hr; Tel.: +385-92-172-1314; 2 Department of Pathophysiology, University of Split School of Medicine, 21000 Split, Croatia; 3 University Department of Health Studies, University of Split, 21000 Split, Croatia

The goal of this Special Issue was to collect original pieces as well as state-of-the-art review articles from scientists and research groups with specific interests in atherosclerosis research. However, why is atherosclerosis important in the first place? The main principles about atherosclerosis onset and natural history have been well recognized throughout the recent few centuries. It merely refers to the chronic accumulation of fatty and/or fibrous material in the intimal layer of arteries that can later progress to obstruct arterial lumen, compromise blood flow, and result in tissue ischemia—depending on which vascular territory is involved [1]. As a systemic disease, atherosclerosis can significantly affect multiple vascular beds, including carotid arteries and cerebral arteries, coronary vessels, thoracic and abdominal aorta, renal arteries, and superior and inferior mesenteric arteries. The onset of these atherosclerotic lesions is facilitated by low-density lipoprotein cholesterol (LDL-c) that is responsible for the transfer of cholesterol within the circulatory system. Although continuous accumulation of lipids and lipid-rich cells plays a central role in atherogenesis, modern conceptions of atherosclerosis deeply involve other mechanisms, such as systemic inflammation through inflammasome activation, clonal hematopoiesis of indeterminate potential, and calcification [1,2]. From a practical standpoint, atherosclerosis remains the sole leading cause of vascular disease worldwide. Moreover, it is the principal mechanism responsible for severe clinical manifestations, such as ischemic heart disease, ischemic stroke, and peripheral artery disease, which all are highly prevalent in global societies [3,4]. In fact, subclinical atherosclerosis was present in as much as 63% of the asymptomatic middle-aged population (71% of men and 48% of women), as shown in the PESA (Progression of Early Subclinical Atherosclerosis) study [5]. Furthermore, atherosclerotic cardiovascular disease (ASCVD) is strongly associated with poor clinical outcomes, such as increased morbidity and mortality, but also imposes a significant economic impact on society and healthcare systems [6]. In reference to all the above-stated reasons, atherosclerosis is a clinical entity that should be perceived as a priority within the global research framework, both in preclinical and clinical terms.

The presented collection of manuscripts delved into novel insights about molecular mechanisms and potential therapeutic targets in atherosclerosis. These include endothelial dysfunction, inflammatory responses, and oxidative stress. In their work, which included 230 patients with established coronary artery disease (CAD), Cuciuc and colleagues investigated the role of the soluble form of the Triggering Receptor Expressed in Myeloid Cells 2 (sTREM2) membrane receptor [7]. As a constituent part of the innate and adaptive immune system, macrophages actively dictate the fate of atherosclerotic plaques as they ingest normal and modified lipoproteins, transforming them into the cholesterol-rich “foam cells” that actively promote atherogenesis [8]. Because sTREM2 is expressed, among other tissues, in atherosclerotic macrophages, authors hypothesized that circulating sTREM2 levels would be associated with cardiovascular outcomes in patients with established CAD in the context of chronic coronary syndrome (CCS). They report that patients in the highest quartile of sTREM2 levels had worst outcomes; this was associated with a multivariate-adjusted hazard ratio of cardiovascular death of 2.37 (95% CI 1.17–4.83, *p*-value of 0.016). Authors provide a putative explanation for their main finding: increased levels of sTREM2 among patients with CCS that suffered cardiovascular death during the follow-up could be mechanistically linked to upregulated innate immunity activation. This is reflected by the greater shedding of sTREM2 from plaque macrophages due to an ongoing process of plaque destabilization and plaque rupture. Indeed, while it is recognized that not all vulnerable plaques will eventually rupture [9], those that rupture are potent drivers of cardiovascular mortality; it was previously shown that such plaques were involved in coronary thrombosis in 73% of all patients that experienced sudden coronary death [10]. It has also been observed that many advanced plaques may undergo several non-fatal ruptures and not cause cardiovascular death [11]. Therefore, it becomes obvious that the direct relationship of plaque morphology and cardiovascular death is a complex and not always intuitive issue. Nevertheless, theoretical propositions provided by the Cuciuc et al. offer a plausible explanation as to why patients with CCS and the highest circulating sTREM2 levels experienced the greatest number of cardiovascular deaths.

By accepting the notion that different coronary plaque phenotypes and mechanisms of plaque destabilization exhibit different natural history and subsequent prognosis, the ability to distinguish between these in clinical practice becomes paramount. For these reasons, Gurgoglione et al. provide a comprehensive, up-to-date review of intravascular imaging (IVI) modalities, which enable us to study and evaluate human coronary plaques in vivo and to understand their impact on patient prognosis [12]. Their work embodies both the diagnostic and treatment aspects of using IVI in modern interventional cardiology practice. This includes intravascular ultrasound (IVUS), optical coherence tomography (OCT), near-infrared diffuse reflectance spectroscopy (NIRS), and other hybrid technologies. They also incorporate modern insights into the pathophysiology of atherosclerosis, the relationship between pathophysiology and imaging features, and define the role of IVI and its therapeutic implications. Finally, they conclude that IVI will play an important role in risk stratification and atherosclerotic plaque characterization among patients with CAD and will dictate the personalized and differential management of these patients. Such conclusions are further validated by the latest research data showing that use of IVI during percutaneous coronary intervention (PCI) and stent implantation is safe and effective and associated with a significant risk reduction of death, myocardial infarction, repeat revascularizations, and stent thrombosis among patients with CAD [13].

One of the most common clinical correlates of overt coronary atherosclerosis is a chest pain (or angina), which significantly impacts patients’ quality of life. However, in many cases, these symptoms might be present without the presence of significant atherosclerotic lesions in epicardial coronary arteries; however, they are instead related to a microvascular disease. Coronary microcirculation is the most potent regulator of coronary blood flow and perfusion, while the epicardial coronary bed merely serves as a conduit for blood flow [14]. In their review published in this Special Issue, Kei et al. provided insights about the heterogenous mechanisms of coronary microvascular dysfunction (CMD) along with its clinical implications and potential treatments [15]. CMD presents an important research area because it was shown that approximately 50% of all patients with anginal symptoms and no obstructive epicardial CAD exhibited CMD and/or coronary spasm [16,17]. These patients are often difficult to treat, and their quality of life is significantly diminished. Therefore, more effort is needed to understand the mechanisms underpinning this relevant clinical entity.
